# Comparison of whole blood and serum samples of breast cancer based on laser-induced breakdown spectroscopy with machine learning

**DOI:** 10.1364/BOE.489513

**Published:** 2023-05-03

**Authors:** Bushra Sana Idrees, Geer Teng, Ayesha Israr, Huma Zaib, Yasir Jamil, Muhammad Bilal, Sajid Bashir, M. Nouman Khan, Qianqian Wang

**Affiliations:** 1 School of Optics and Photonics, Beijing Institute of Technology, Beijing 100081, China; 2 Key Laboratory of Photonic Information Technology, Ministry of Industry and Information Technology, Beijing Institute of Technology, 100081 Beijing, China; 3 Department of Engineering Science, Institute of Biomedical Engineering, University of Oxford, Oxford, OX3 7LD, United Kingdom; 4 Laser Spectroscopy Lab, Department of Physics, University of Agriculture Faisalabad, 38090, Pakistan; 5 Institute of Engineering Thermophysics, Chinese Academy of Sciences, Beijing 100190, China; 6 University of Chinese Academy of Sciences, Beijing 100049, China; 7 Punjab Institute of Nuclear Medicine Hospital, Faisalabad 2019, Pakistan; 8 Yangtze Delta Region Academy of Beijing Institute of Technology, Jiaxing 314033, China; 9 geer.teng@eng.ox.ac.uk; 10 qqwang@bit.edu.cn

## Abstract

To identify cancer from non-cancer is one of the most challenging issues nowadays in the early diagnosis of cancer. The primary issue of early detection is to choose a suitable type of sample collection to diagnose cancer. A comparison of whole blood and serum samples of breast cancer was studied using laser-induced breakdown spectroscopy (LIBS) with machine learning methods. For LIBS spectra measurement, blood samples were dropped on a substrate of boric acid. For the discrimination of breast cancer and non-cancer samples, eight machine learning models were applied to LIBS spectral data, including decision tree, discrimination analysis, logistic regression, naïve byes, support vector machine, k-nearest neighbor, ensemble and neural networks classifiers. Discrimination between whole blood samples showed that narrow neural networks and trilayer neural networks both provided 91.7% highest prediction accuracy and serum samples showed that all the decision tree models provided 89.7% highest prediction accuracy. However, using whole blood as sample achieved the strong emission lines of spectra, better discrimination results of PCA and maximum prediction accuracy of machine learning models as compared to using serum samples. These merits concluded that whole blood samples could be a good option for the rapid detection of breast cancer. This preliminary research may provide the complementary method for early detection of breast cancer.

## 1. Introduction

More than 2.3 million cases and 685,000 mortalities from breast cancer were reported in 2020. Australia/New Zealand, Western Europe, Northern America, and Northern Europe had the highest incidence rates (>80 per 100,000 females), whereas Central America, Eastern and Middle Africa, and South-Central Asia had the lowest rates (<40 per 100,000) [1,2,3]. By 2040, the problem of breast cancer is estimated to increase to over 3 million new cases and 1 million fatalities every year due to population growth and aging alone [3]. In the U.S. breast cancer affects over 13,000 women every year, who are 40 years old or younger. Furthermore, among women aged 20-39, breast cancer ranks first in terms of cancer-related deaths [4]. Today, a breast cancer diagnosis is still a major challenge in clinical medicine. According to the U.S. National Cancer Institute, the cost of diagnosing and treating breast cancer may exceed $20.5 billion per year [5]. Nowadays, a variety of methods are being used for the screening of breast cancer, including in-line X-ray synchrotron phase contrast tomography (XSPCT), magnetic resonance imaging (MRI) [6], microwave imaging technique [7], fluorodeoxyglucose (FDG) positron emission tomography/computerized tomography (PET/CT) [8] and Raman spectroscopy [9]. These breast cancer detection techniques have limitations such as high costs, harmful radiation, poor resolution, sensitivity, and inconveniences to the patients [10]. Mammography is known as a gold standard technique for breast cancer screening, but it is not suitable for patients under 40 years old and with dense breasts, less sensitive to tumors (smaller than 1 mm, approximately 100,000 cells), and does not indicate prediction about eventual disease outcome. Moreover, the ionizing radiation from X-rays increases the risk of cancer in women who use mammography as a screening test [11,12]. Improved survival rates depend on early identification and rigorous diagnostic procedures. Although the current method for diagnosing Triple Negative Breast Cancer (TNBC) from the histopathology of biopsy samples is highly accurate, it still has some drawbacks that limit its use, including a complicated procedure, time taking, invasiveness, and the requirement for professionals [13,14]. Therefore, it is necessary to find experimental techniques with high sensitivity, speed, economy and robustness for early-stage illness identification before the beginning of micro metastases and to decrease the fatality rate among women. In clinical diagnosis, the blood is a standard sample form and must be collected for other measurements. Meanwhile, cancer induces some dissociated particles in the blood and causes subtle composition changes [15]. Therefore, if combined with an appropriate and successful information extraction technique, analyzing the atomic information of blood might be an effective solution to meet the above-mentioned need.

Laser-induced breakdown spectroscopy (LIBS) is a useful technique because it can rapidly identify multiple elements with high resolution [16,17]. Specific wavelength-related spectral lines reflect the information about the respective element. The elements distribution can be examined by mapping the sample surface [18,19]. Visual elemental imaging of mice breast cancer tissues using LIBS was carried out to initially understand anti-tumor mechanisms. The analysis of four distinguishable elemental calcium (Ca), copper (Cu), magnesium (Mg) and sodium (Na) from tumor tissues showed the therapeutic effects of drugs on the tumor [6]. It is challenging to get samples of human biological tissue due to medical ethics. Researchers have started to consider using samples obtained during routine tests, such as blood, to introduce this technology to clinical investigations in terms of practical applicability.

LIBS combined with machine learning plays a remarkable role in the diagnosis of different malignancies. Machine learning is a modern data mining method established by artificial intelligence (AI) and more efficient for extracting the appropriate distinguishing characteristics from a sample [20]. AI has recently been utilized to identify COVID-19 positives rapidly by using CT imaging technology and related clinical data. Radiologists identified all of these patients as COVID-19 negative through reverse transcription- polymerase chain reaction (RT-PCR) and CT scans, but the AI system correctly recognized 17 of 25 patients (68%) as COVID-19 positive with significant enhancement [21]. The ability of related algorithms for classification and identification, containing Support Vector Machines (SVM), Gradient Boosting Linear Discriminant Analysis (LDA) and Fisher Discriminant Analysis (FDA) with accuracy up to 96% were fully demonstrated by advances in medicine for the diagnosis of melanoma in biomedical fluids (tissue and blood homogenates) dropped on the solid substrate [22]. In a recent study, Xue Chen et al. combined the LIBS technique with chemometric methods to discriminate the whole blood samples of lymphoma patients with healthy samples by using principal component analysis (PCA), k-nearest neighbor (*k*-NN) and LDA models. With an accuracy of over 99.7%, 99.6% sensitivity and 99.7% specificity, both LDA and *k*-NN models showed good discrimination analysis [15]. Y. Chu et al. illustrated that Nasopharyngeal Carcinoma (NPC) and healthy serum samples can be distinguished using LIBS in combination with a random forest-extreme learning machine (RF-ELM) model to identify NPC accurately. The identification accuracy rate, sensitivity, and specificity of NPC serum and healthy samples achieved 98.3%, 99% and 99.7%, respectively [23]. This study demonstrates that machine learning techniques paired with blood sample-based LIBS can be a non-invasive, fast and reliable diagnosis technique for human malignant tumors.

Indeed, lots of research have been conducted on this subject, the precise method to investigate the concentration of elements in the blood by different malignancies is still not clear [24,25]. The concentration of trace elements in the blood plays a crucial role in a variety of biological processes by activating or inhibiting enzymes, metalloproteins for binding sites, interacting with other elements and changing the permeability of cell membranes [24]. It is usually considered that any changes in the human body might result in changes in the blood because blood serves as the medium for the movement of trace components [25]. Changes in the amounts of trace elements are either the cause or a consequence of improper metabolism and the growth of malignancies.

Although, some other works have been conducted LIBS in blood analysis [26]. The novelty in our work is that the difference of whole blood and serum samples in LIBS breast cancer diagnosis. We proposed an efficient and fast method for early diagnoses of human breast cancer with a comparison of whole blood and serum samples using LIBS combined with machine learning models. After depositing on the boric acid substrate, the effects of two kinds of samples were compared during experimental measurements to investigate the better type of sample. Classification models including the decision trees (DT), discriminant analysis (DA), logistic regression (LR), naive Bayes (NB), *k*-NN, support vector machines (SVM), ensembles classifiers (ECS) and neural networks (NN) classifier were used to compare the classification results of whole blood and serum samples for the diagnosis of breast cancer.

## 2. Materials and methods

### 2.1 LIBS experimental setup

**Fig. 1.**
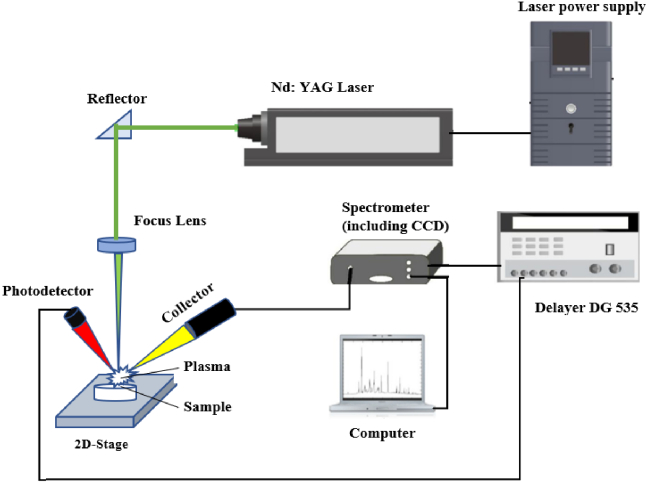
Schematic of LIBS Experimental Setup.

In the experiment, a Q-switched Nd: YAG laser (Q-smart 850) operating at 10 Hz was employed with a second harmonic (wavelength 532 nm) at a pulse energy of 103 mJ/pulse of diameter Ø6 mm focus through a biconvex lens of focal length 115 mm to initiate laser plasma in the blood samples. The plasma emission is collected into an optical fiber spectrometer (AvaSpec 2048–2-USB2, Avantes) with a range of 190 nm to 770 nm and a resolution of 0.08 nm and detected by the charge-coupled device (CCD). A delay generator (DG535, Stanford Research Systems) was used to adjust the timing of the triggering pulse for the Q-switch and spectrometer. Figure 1 shows the LIBS experimental setup for the detection of breast cancer. The delay time for the spectrometer to be triggered was set at 1 µs, and the integration time of the CCD was set at 2 ms. A two-dimensional translation stage was used to adjust the laser spot position on the samples. This experiment was carried out in normal air conditions.

### 2.2 Whole blood and serum samples

**Fig. 2.**
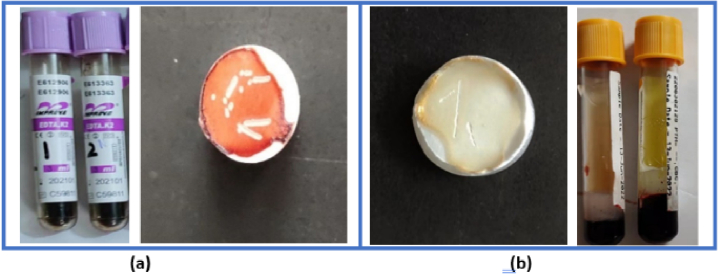
The image of (a) whole blood samples stored in EDTA tubes and on a boric acid substrate and also (b) serum samples stored in SST and on a boric acid substrate after ablation.

In this work, 13 whole blood cancer samples and 11 whole blood healthy samples were collected. Every sample was from a different patient or volunteer, and were examined by Punjab Institute of Nuclear Medicine (PINUM) cancer hospital, Pakistan. Similarly, 18 serum cancer samples and 20 non-cancers samples were also collected from all different individuals for analysis. All the cancer samples including whole blood and serum belongs to the stage III C based on previously diagnosed by pathological examination of biopsy samples of tissue and lymph nodes. The clinical protocol for both types of samples was certified by the Clinical Research Ethics Committee of PINUM Hospital. Whole blood samples were obtained from the vein inside the individual's arm near the elbow and placed in ethylenediaminetetraacetic acid (EDTA) tubes to prevent blood clotting. Serum samples were obtained after centrifugation in a serum separator tube (SST) which contains separating gel and clot activator to create a barrier between whole blood and serum. After collection, the samples were kept in a 4 °C refrigerator until the LIBS measurement, which was carried out within 48 hours of sample collection. Before LIBS analysis of liquid samples, the liquid blood samples were solidified for enhancing the spectral signal. Figure 2 shows (a) whole blood samples stored in EDTA tubes and on a boric acid substrate and similarly (b) serum samples store stored in SST and on a boric acid substrate after ablation. The pre-treatment steps are given below:

(1) A micropipette was used to pour 50 µl of blood sample onto a pellet for each sample. Each pallet was made by pressing 99.7% pure boric acid powder at 20 Mpa in a hydraulic press, and the pellet diameter is about Ø40 mm.
(2) Each blood sample was dried in the air for more than 5 minutes.
(3) For each sample, 60 spectra were collected, each on a fresh position. The focus position of the laser on the sample was adjusted by a 2D motorized stage. The point of focus changed with each laser shot. If we repeat the laser shots in the same position, the crater formed before will cause a disturbance in the LIBS signals.

## 3. Data processing procedure and methods

Classification between whole-blood samples of breast cancer and non-cancer is represented in case 1 and the discrimination between serum samples of breast cancer and non-cancer is in case 2.

### 3.1 Data preprocessing

The following processes were included in data preprocessing.

(1) Averaging process: 60 raw spectra of each cancer and non-cancer samples were obtained from both whole blood and serum samples. Total number of raw spectra from whole blood samples of cancer was 780 and non-cancer was 660. Similarly, the total number of raw spectra from serum samples of cancer was 1080 and non-cancer was 1200. To minimize spectral fluctuations caused by laser pulse shot-to-shot energy fluctuation and inhomogeneity of samples, calculated an average spectrum of every five adjacent raw spectra, total 12 averaged spectra were obtained from each sample in both cases. Total 156 and 132 averaged spectra from whole blood samples of breast cancer and non-cancer, and 216 and 240 averaged spectra from serum samples of breast cancer and non-cancer were obtained, respectively. Table 1 listed the distribution of samples and spectra for both cases.
(2) Normalization: Each average spectrum was normalized with its total spectral intensity calculated by integrating the spectral intensity over the whole spectral range. The preprocessed spectra were produced by the preceding process.

**Table 1.** Description of the samples and their spectra for case 1 and case 2

Case No.	Sample Type	No of sample	Total no of raw spectra	Total no of averaged spectra
Case 1.(Whole blood)	Cancer	13	780	156
	Non-Cancer	11	660	132
Case 2. (Serum)	Cancer	18	1080	216
	Non-Cancer	20	1200	240

### 3.2 LIBS spectral analysis

**Fig. 3.**
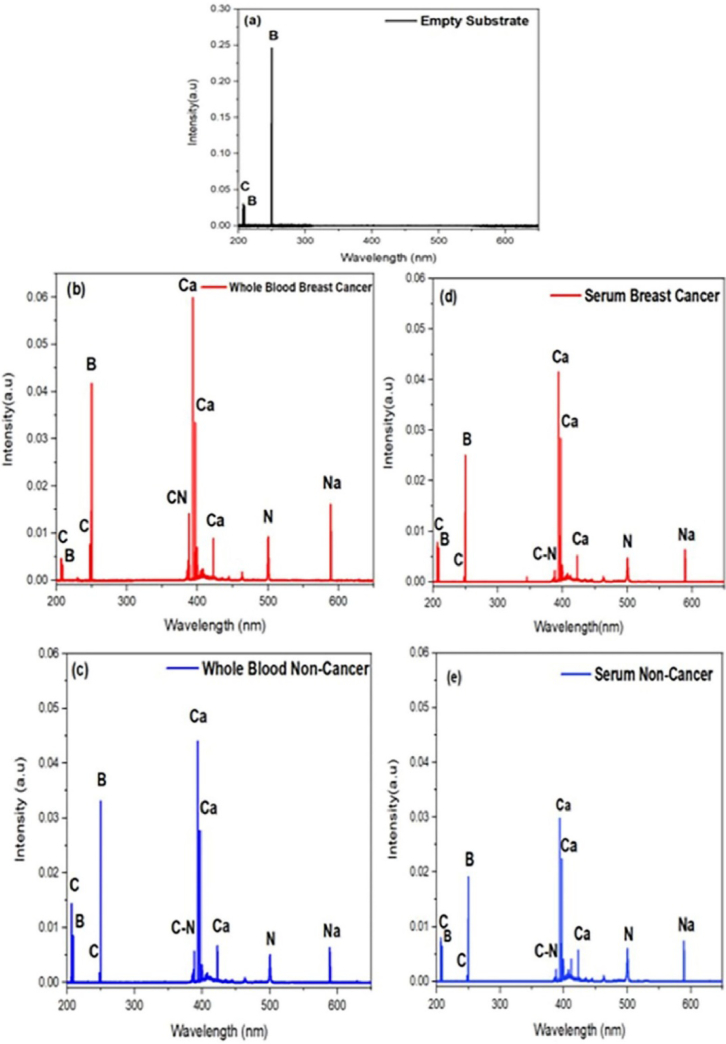
LIBS spectra of average normalized intensities of several atomic emission lines of the (a) substrate, (b) and whole blood breast cancer with (c) non-cancer samples, and (d) Serum breast cancer with (e) non-cancer samples in the spectral range 200-650 nm.

The LIBS spectra of the substrate, whole blood samples of breast cancer and non-cancer as well as serum samples of breast cancer and non-cancer are shown in Fig. 3. Significant emission lines of boron (B) and carbon (C) can be seen in the average spectrum of a substrate. Breast cancer and non-cancer samples have quite different spectra than the substrate. The atomic emission lines in these spectra were labeled by using NIST atomic emission database [27]. Here, the strong spectral lines of Calcium (Ca), Nitrogen (N), Sodium (Na), CN-band and C were noticed in both breast cancer and non-cancer samples. The concentration of these elements in cancer samples are greater than those in non-cancer samples.

In this work, the materials of substrate for the blood samples were taken into consideration. The substrate with single component and minimal interference to blood samples was used in this analysis. Chu et. al. also used boric acid pallets for the early diagnosis of blood cancer samples. Elements including Ca, Mg, Na, K and CN-band with different concentrations were observed in the blood cancer samples and only B, O, N and H were observed in the empty boric acid pallet of LIBS spectral analysis [28]. Here, the significant emission lines of Na, Ca, N, C and CN-band were existed in the spectra of breast cancer samples and only B and C lines were found on the LIBS spectra of empty boric acid pallet and was not used in the selected lines of analysis. Boric acid is used as a substrate because of a smaller number of elements and less emission lines were shown in LIBS spectra of empty substrate pallet to reduce the influence of elements in the blood sample.

For the discrimination analysis, 11 emission lines were chosen, including 7 atomic lines of Ca, C, Na, N and 4 from the CN-band. The prominent characteristic emissions with higher concentrations of Ca, Mg, Na, C_2_ and CN-band were observed in the LIBS spectra of tissue samples of breast cancer patients compared to the healthy one [29]. As C and N are the main components in a biological cell. Both endogenous and exogenous factors were associated to the C and N emission lines. Na is another potential biomarker, and its abundance rises because of the cancer. In physiological saline, Na plays a significant role in regulating the balance of physiological regulation. CN-bands may result from the ablation of CN-bands in amino acid structures within the cells or from the recombination of C components stimulated from the sample and N in the surrounding atmosphere [30]. The Ca regulates a wide range of cellular functions, including gene transcription, cell proliferation and muscle contraction, it is almost a universal intracellular messenger. On the other hand, the ducts that carry the milk and the glandular tissue where milk is produced both experience calcification. This effect, which is frequent in lobules, is usually benign. Sometimes, the ductal calcification is a sign of preinvasive ductal carcinoma in situ (DCIS), which is noninvasive. A calcified track appears along the duct's path when the tumorous cells in the middle of ducts die (mostly as a result of the lack of nutrients) [31]. The concentration of Ca is more important than other elements for also the identification of many other malignancies. Excess of Ca lines with high intensities in Gastrointestinal stromal tumor (GIST) tissues in comparison to its healthy tissues is related to the presence of tumor [32]. It may also show great distinguishing ability in breast cancer detection. El-Hussein et al. have reported higher Ca and Mg concentrations in breast and colorectal cancer tissues in contrast to non-neoplastic ones [33]. Table 2 lists the details of the selected lines based on the NIST database. The spectral emission lines of the boric acid pallets are not subtracted because their intensities were not much higher than those of the blood samples and were stable during the experimental measurements. The concentration of Ca and other elements decreases in the spectra of serum samples, it may be because of the centrifugation of blood. Meanwhile, emission lines of Ca, C, N, Na and CN-band were observed in both types of samples, but whole blood samples contained strong emission lines as compared to serum samples because of enhancement in signal-to-noise (S/N) ratio as compared to serum samples spectra.

**Table 2.** 11 atomic emission lines with corresponding elements

Elements	Wavelength (nm)
C	247.8
Ca	393.4, 396.8, 422.7
CN	385.7, 386.19, 387.1, 388.3
N	500.5
Na	588.9, 589.5

**Fig. 4.**
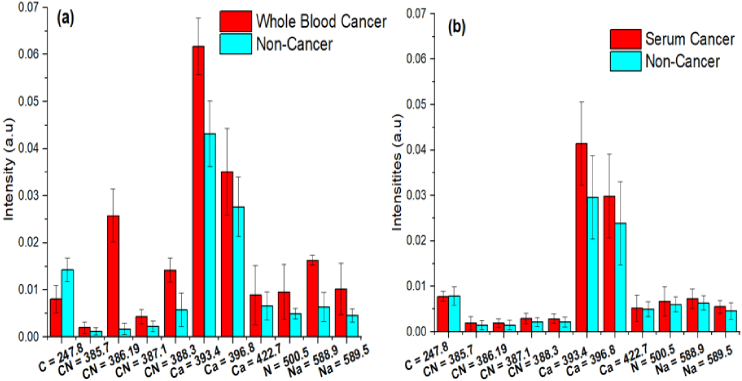
Histogram comparing the average normalized intensities of 11 LIBS emission lines of (a) whole blood and (b) serum samples of both cancer and non-cancer samples. The error bars are the standard deviations of the intensities of independent spectra.

In order to examine the uncertainties in the spectral data, histogram was used for both cases. Histogram comparing the average normalized intensities of 11 LIBS spectral lines of both cancer and non-cancer samples. The error bars are the standard deviations of the intensities of independent spectra for both cases are shown in Fig. 4. Mean percentage of fluctuation in the intensities of these lines was less than 0.5% in both cases. Almost all the emission lines of Ca, Na, N, C and CN-band in histogram show higher intensities of breast cancer samples than non-cancer samples Enhancement in the concentration of Ca lines may provide the higher discrimination in breast cancer detection.

## 4. Results and discussion

### 4.1 Discrimination analysis

**Fig. 5.**
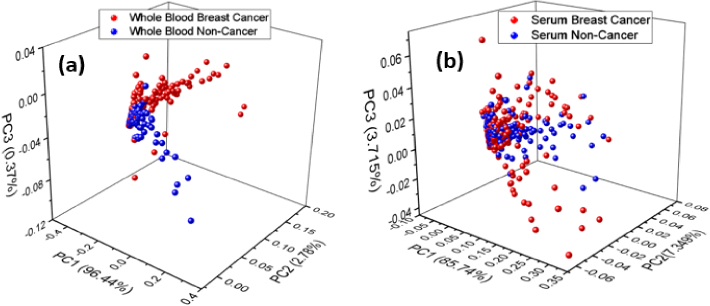
PCA clustering analysis of (a) whole blood and (b) serum samples of breast cancer and non-cancer.

**Fig. 6.**
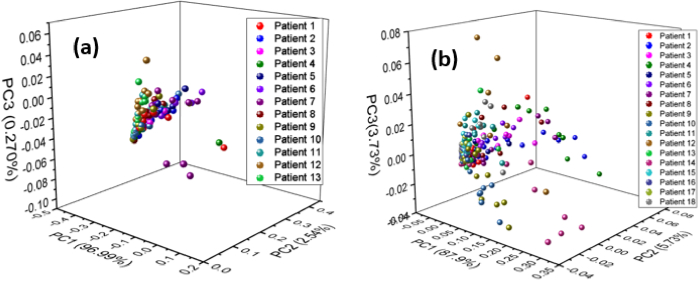
PCA clustering analysis of each patient of breast cancer samples of (a) whole blood (b) serum.

The principal component analysis (PCA) was implemented as an unsupervised machine learning method on the spectral data matrix to reduce the dimensionality and to distinguish between breast cancer and non-cancer with both types of samples. Figure 5 shows the PCA clustering analysis of (a) whole blood and (b) serum samples of breast cancer and non-cancer. The cumulative variance was primarily defined by the first three principal components (PCs). i.e., 99.5% in case of whole blood samples and 96.8% in case of serum samples, respectively. The PCA results of whole blood and serum samples of breast cancer and non-cancer were not well classified. Meanwhile, the whole blood samples show better discrimination in comparison to serum samples because of dispersion of points in each region of cancer and non-cancer samples is relatively large. Furthermore, due to overlapping the scores of cancers and non-cancer samples are not within their respective regions in both cases. Figure 6 shows the clustering analysis based on the first three PCs. (a) Taking all 156 averaged spectra of whole blood breast cancer samples and (b) 216 averaged spectra of serum breast cancer samples into the PCA. It can be seen that the sample spots found in the various breast cancer samples were mixed and could not be completely differentiated. This also showed that the elemental components of breast cancer patients were normally consistent. Data preprocessing method was used to minimize interpatient variability among the patients. For this purpose, averaged and normalized spectral data were used to weaken the influence of variation within each patient. The LIBS spectra obtained from breast cancer samples have good stability although each patient has certain individual differences. This supports the potential of LIBS as a possible breast cancer detection technique.

### 4.2 Multivariate statistical analysis

#### 4.2.1 Machine learning methods

For the classification of breast cancer and non-cancer samples, machine learning models were examined to improve the identification feature of LIBS. In case 1, among 13 breast cancer samples, 9 samples with 108 averaged spectra were randomly selected as training set and 4 samples with 48 averaged spectra were left for prediction set. Among the 11 total number of non-cancer samples, 8 samples with 96 averaged spectra were randomly selected as training set and the other 3 samples with 36 averaged spectra were used for prediction set. There was no overlapping between the training and prediction sets from the individual perspective. In the same way, the distribution of samples with their spectra for training and predicting were selected for case 2. 12 cancer ones with 144 averaged spectra and 13 control ones with 156 average spectra were randomly selected for the training, and 6 cancer ones with 72 average spectra and 7 control ones with 84 averaged spectra were left for the prediction in case 2. In both cases all the samples with their related spectra were selected for training or prediction set, therefore, there is no correlation between the spectra of training and prediction set. The distribution of prediction and training datasets of samples and their spectra in both cases is shown in Table 3.

**Table 3.** Distribution of training and prediction sets for case 1 and case 2

Data Separation		Whole Blood (Case. 1)		Serum (Case. 2)	
		Cancer	Non-Cancer	Cancer	Non-Cancer
**Training Set**	**No of Samples**	9	8	12	13
	**No of Spectra**	108	96	144	156
**Prediction Set**	**No of Samples**	4	3	6	7
	**No of Spectra**	48	36	72	84

DT, DA, LR, NB, *k*-NN, SVM, ELC and NN are a few supervised machine learning methods that have been used in classification analysis. A lot of research has been done by using all these algorithms for the identification of breast cancer samples with different methods [34].

A decision tree is a classification model that is employed for both regression and classification. Making decisions is guided by a tree structure from the root to the final class (leaves). DT is constructed by analyzing a dataset of training samples for which the class labels are already known. Furthermore, if the DT model is trained on good-quality data, it can produce maximum accuracy of prediction dataset [35]. Classification And Regression Tree (CART) method was used here to distinguish between cancer and non-cancer samples. The Gini impurity index is employed to find the probability of incorrect classification. Fine tree, medium tree and coarse tree are the tested variants. The maximum number of splits used was 100 for a fine tree, 20 for a medium tree and 4 for a coarse tree. These were used to compare the accuracy of every variant.

Discriminant analysis is used to differentiate the information of two or more classes depending on the supposition that various classes have distinct multivariate normal distributions of the predictor features. For the linear discriminant analysis (LDA), the multivariate normal distributions have the same covariance matrix for each class, and only the means differ. Both the mean and covariance matrix change for each class in quadratic discriminant analysis (QDA), which produces more versatility. To find the subscription of an unknown sample, the DA model computes the posterior probabilities that the sample is related to various classes. The sample is then attributed to the class with the highest posterior probability [36]. For the identification of breast cancer samples both LDA and QDA were examined. Feature lines were used as a predictor in the discriminant analysis. This was performed to decrease the dimensionality of the dataset and prevent overfitting.

The link between a dichotomous dependent variable and several independent variables that are either categorical or continuous is modeled using binary logistic regression. Binary logistic regression has various presumptions that must be met to produce a reliable result. A variety of logistic regression remedies include enlarging the sample size, eliminating one of the correlated variables, and integrating variables into an index. Despite increasing sample size, it may be reliably inferred that leaving out one of the correlated variables can significantly lessen multicollinearity [37]. This is especially helpful when the sample size is small or the classes have comparable variance structures. Here, binary logistic regression as a function of a linear combination of predictors was used to classify cancer and non-cancer samples.

A Naïve Bayes classifier follows the Bayes theorem (from Bayesian statistics) and strong (naive) independence assumptions. The main benefit of this classifier is that it only needs a small amount of training data to calculate the means and variances of variables compulsory for discrimination. It is not necessary to calculate the entire covariance matrix because independent variables are supposed. Only the variances of the variables for each label must be calculated [38]. Here, Kernel Naïve Bayes used kernel distribution and Gaussian Naïve Bayes used Gaussian distribution for predictors was examined.

SVM model follows the principle of dimension reduction and the Vapnik-Chervonenkis dimension theory of statistical learning. The objective of SVM is to identify the accurate classification hyperplane to satisfy discrimination requirements, which increased the blank area on both sides of the hyperplane while verifying the accuracy of classification [39]. Here, the optimized kernel scale for linear SVM was 1, quadratic SVM was 1, cubic SVM was 1, fine SVM was 1.5, medium SVM was 6 and coarse SVM was 24 optimized in this study.

*k*-NN is employed in pattern recognition and discrimination methods. A Euclidean distance measure between specific point “x” and known training dataset points in the prediction dataset, where taking k known training dataset points by shortest distance from a specific point and determining the value of k associated with which class, determining the class with the maximum classification and assigning the “x” point to that class [40]. The number of neighbors for Fine *k*-NN was 1, Medium *k*-NN was 10, Cubic *k*-NN was 10, Coarse *k*-NN was 100, Cosine *k*-NN was 10 and Weighted *k*-NN was 10 used in this study to compare the accuracies of both cases.

An ensemble classifier is a method that uses many classifiers to solve a specific problem using a specific combination rule [41,42]. Conceptually, the single method that contains the ensemble is designed to solve the same problem individually. The final output of the ensemble is the sum of the outputs of the single method. There are two types of ensemble methods: homogenous and heterogeneous ensembles. Homogeneous ensembles belong to two potential subtypes: (1) a combination of a meta-ensemble method such as boosting, bagging and random subspace and one single method and (2) a combination of at least two variants of the same machine learning method, whereas heterogeneous ensembles are a combination of at least two distinct machine learning methods [43]. The primary goal of each ensemble classifier is to achieve high accuracy. This work investigates the different types of ensemble classifiers, such as subspace discriminant and subspace *k*-NN, as well as boosted trees, RUS boosted trees, bagged trees, and subspace trees, in breast cancer detection. The maximum number of splits for each classifier was 20.

A neural network model contains a group of models that find out underlying correlations in a group of data using a method related to how the human brain works. In this concept, neural networks are groups of neurons that may have a biological or artificial origin. The linked components of the decision regions are unbounded for neural network functions with a maximum width less than or equal to the input dimension. Hence, the decision regions of such networks intersect with the boundary of a natural input domain. [44]. Here, a neural network is used to compare the accuracies of their different classifiers including narrow NN, wide NN, medium NN, bilayer NN and trilayer NN. Rectified Linear Unit (ReLU) used as activation function. The size of one completely connected layer was 10 for narrow NN, 25 for medium NN and 100 for wide NN. Each two-connected layer size in Bilayer NN was 10 and in trilayer NN each three-connected layer size was 10. Furthermore, a detailed description of all the above classifiers is in the given Refs. [35,44].

The spectral line intensities of breast cancer and non-cancer were different, but it was hard to discriminate by examining directly as well as through the PCA. Machine learning methods were combined to enhance the performance of LIBS to distinguish breast cancer and non-cancer samples.

#### 4.2.2 Comparison of classification results

The discrimination models were trained and predicted (tested) using two cases. Whole blood samples discrimination against breast cancer and non-cancer has been done in case 1. In case 2, machine learning models discriminate the serum samples of breast cancer and non-cancer. A 10-fold cross-validation approach was used as a preventative measure against overfitting. Function parameters of all the models were optimized by 10-fold cross validation and a grid search based on training dataset. The optimal parameter combination leading to the best cross validation result was used to build the classification model. Table 4 shows the classification results of case 1 and case 2 with optimized parameters and functions of each model.

**Table 4.** Classification results of case 1 and case 2

Classifier	Classifier Type	Optimized Parameter		Function	Case 1. Distinguish Whole Blood (Cancer Vs Non-Cancer)		Case 2. Distinguish Serum (Cancer Vs Non-Cancer)	
					Training Accuracy (%)	Prediction Accuracy (%)	Training Accuracy (%)	Prediction Accuracy (%)
**Decision Trees**	Fine Tree	No of splits	100	Gini’s diversity index	97.1	81.0	95.0	89.7
	Medium Tree		20		97.1	81.0	95.0	89.7
	Coarse Tree		4		97.1	81.0	95.0	89.7
**Discrimination Analysis**	Linear Discrimination	Scale	1	Linear	88.2	90.5	92.0	66.7
	Quadratic Discrimination		1	Quadratic	90.2	82.1	94.0	75.6
**Logistic**	Regression		1	Linear	89.2	77.4	92.7	70.5
**Naïve Byes**	Gaussian Naïve Bayes	Scale	1	Gaussian	93.6	69.0	91.0	74.4
	Kernel Naïve Bayes		1	Kernel	92.2	72.6	85.7	44.2
**Support Vector Machine (SVM)**	Linear SVM	Kernel Scale	1	Linear	90.2	76.2	94.7	69.9
	Quadratic SVM		1	Quadratic	91.7	82.1	93.7	64.7
	Cubic SVM		1	Cubic	96.1	82.1	90.7	64.7
	Fine Gaussian		1.5	Gaussian	84.3	86.9	87.7	48.1
	Medium Gaussian		6		86.8	86.9	91.7	67.9
	Coarse Gaussian		24		74.5	86.9	73.7	60.3
**K Nearest Neighbor (*k*-NN)**	Fine *k*-NN	No of Neighbors	1	Euclidean	88.2	83.3	90.7	64.7
	Medium *k*-NN		10		81.4	88.1	87.0	59.6
	Coarse *k*-NN		100		68.6	84.5	69.3	59.6
	Cosine *k*-NN		10	Cosine	83.3	86.9	88.7	59.0
	Cubic *k*-NN		10	Minkowski	80.9	90.5	85.3	60.3
	Weighted *k*-NN		10	Euclidean	86.3	86.9	88.3	60.3
**Ensemble Classifier**	Boosted Trees	Max. No of splits	20	AdaBoost	52.9	57.1	52.0	53.8
	Bagged Trees		20	Bag	96.1	67.9	96.0	84.6
	Subspace Discriminant		20	Subspace	86.3	69.0	91.7	64.1
	Subspace k-NN		20	Subspace	92.2	69.0	86.0	51.9
	RUS Boosted Trees		20	RUS Boost Bag	79.9	57.1	69.0	53.8
**Neural Network**	Narrow Neural Network	Layer size	1^st^ 10	ReLU	92.2	91.7	95.3	69.9
	Medium Neural Network		1^st^ 25		91.7	86.9	96.7	67.3
	Wide Neural Network		1^st^ 100		94.6	88.1	96.0	69.9
	Bilayer Neural Network		1^st^102^nd^ 10		93.1	87.3	95.3	70.8
	Trilayer Neural Network		1^st^102^nd^ 103^rd^10		92.2	91.7	95.7	71.2

The classification accuracy of each model was calculated as the percentage of the correctly predicted number of spectra to the total number of spectra. To implement classification models, the MATLAB Classification Learner App was used. On MATLAB 2021a, all classification models including their accuracies were applied on this App. The PC used for the calculations had an Intel Core i7-6600U processor unit at 2.60 GHz, Windows 10, and 16 GB of RAM. On MATLAB 2021a, all classification models were applied.

In case 1. when looking at a variety of discrimination models, it was discovered that most classification algorithms could make accurate predictions with an accuracy in the range of 57.1% to 91.7%. Further, it was found that the narrow and trilayer NN calculated the highest prediction accuracy. LDA also showed good results with 88.2% training and 90.5% prediction accuracy. LDA and NN models provided the best option for training and class prediction. For real-world applications, prediction accuracy played an important role in discrimination to examine the model stability. However, narrow NN and trilayered NN both provide 92.2% training and 91.7% prediction accuracy. Indeed, the best model should have the highest prediction accuracy under the premise of balance between training and prediction set. These outcomes demonstrate the variations between the spectra of the same type of blood due to the individual differences between cancer and non-cancer samples. Here, DT, DA, LR, NB, *k*-NN, SVM, ESC’s, medium, wide and bilayer NN produced low prediction accuracy, possibly because of significant overfitting and a certain amount of overlap of data which is intrinsic to input variables. Narrow and trilayer NN could overcome this problem.

**Fig. 7.**
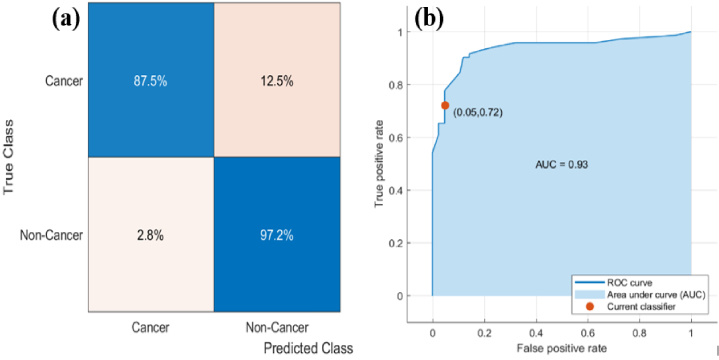
Shows (a) the confusion matrix and (b) the ROC curve of the narrow NN produced from the prediction model for discriminating the whole blood samples of breast cancer versus non-cancer.

In medical diagnosis, a statistical test called sensitivity is used to correctly identify cancer samples, also known as true positive rate, whereas a test called specificity is used to identify non-cancer samples, also known as true negative rate. The confusion matrix was used to determine the specificity and sensitivity of all the models. A confusion matrix is a comparison between predicted class and true class; truly predicted values are located along the diagonal of a matrix. However, false predictions are outside of the diagonal. Confusion matrix and receiver operating characteristic (ROC) results of narrow NN and trilayered NN have a not big difference because of the same accuracy. Therefore, the sensitivity and specificity of only the narrow NN were discussed and calculated from the confusion matrix and ROC curve. In case 1, in the prediction dataset, the narrow neural network achieved the highest classification accuracy at 91.7%. A false positive rate of 12.5% of cancer samples were correctly classified and only 6 spectra showed variation from a single misclassified sample. However, a false negative rate of 2.8% of non-cancer samples were identified as cancer samples and only 1 spectra showed variation from a single misclassified sample. It resulted in a specificity of 97.2% and sensitivity of 87.5% respectively. All cancer and non-cancer samples were completely classified. Only one cancer was mistakenly identified, and one non-cancer was misclassified as cancer. In Fig. 7. (a) confusion matrix and (b) ROC curve of the narrow neural network was produced from the prediction model for discriminating the whole blood samples. To discriminate whole blood samples, the ROC curve depicts true positive value versus false positive value with an AUC (Area under the curve) of 0.93. The working points are indicated on ROC curves in the upper-left corner.

In case 2: The majority of classification models offer prediction accuracy levels of 44.2% to 89.7% when performing to distinguish between serum samples of breast cancer and non-cancer. While a fine tree, medium tree and coarse tree provided a maximum of 95.0% training accuracy and 89.7% prediction accuracy. Bagged trees from the ensemble classifier also showed good results with 96.0% training and 84.6% prediction accuracy. DT and bagged trees gave the best option for training and class prediction. The greatest option for predicting classes was provided by decision tree models. The discrimination between serum cancer and non-cancer samples was accurately predicted. Discriminant models such as LR, NB, *k*-NN, SVM and ESCs except for bagged trees and NN models were not robust enough to produce good prediction accuracy due to the small number of training samples, which seems quite small with the large variations of human serum samples. Despite the precautions used in the data organization into the training and prediction sample sets, the representability of the prediction samples by the training samples cannot, therefore, be guaranteed in an optimized way. For decision tree classifiers, the discrimination results of the training dataset were consistent with the prediction set data. However, the highest prediction accuracy for serum samples is slightly less than whole blood samples, because outliers are found in the spectral data may be because of the centrifugation of blood during the extraction of serum.

**Fig. 8.**
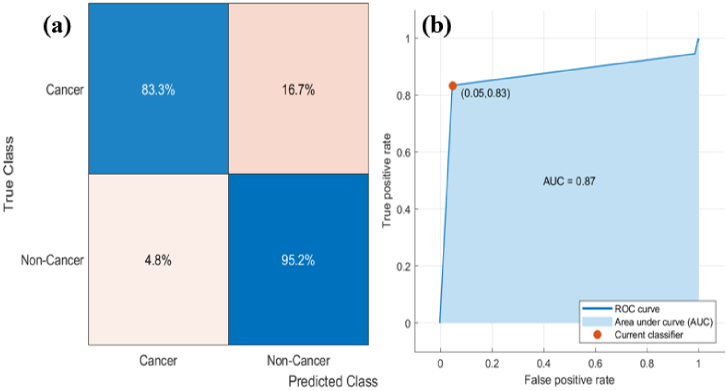
Shows (a) the confusion matrix and (b) the ROC curve of the fine tree produced from the prediction model to distinguish between serum samples of cancer and non-cancer.

The confusion matrix and ROC results of a fine tree, medium tree and coarse tree do not have a big difference because of the same accuracy. Therefore, the sensitivity and specificity of only the fine tree were discussed and calculated from the confusion matrix and ROC curve. In case 2, For the prediction dataset fine tree model achieved the highest classification accuracy at 89.7%. A false positive rate of 16.7% of cancer samples were correctly classified and only 4 spectra from first and 8 from second sample showed variation from misclassified samples. However, a false negative rate of 4.8% of non-cancer sample were identified as cancer sample and 4 spectra showed variation from a single misclassified sample. It resulted in a specificity of 95.2% and sensitivity of 83.3%. All cancer and non-cancer samples were completely classified. Two cancer sample was mistakenly recognized, and only one non-cancer was misclassified as cancer. Figure 8 shows (a) the confusion matrix and (b) the ROC curve of the fine tree produced from the prediction model to distinguish between serum samples of cancer and non-cancer. The ROC curve plots true positive value versus false positive value with the area under the curve (AUC) were 0.87 to distinguish serum samples of breast cancer versus non-cancer.

In this paper, a total of 30 sub-classifiers were built to classify breast cancer and non-cancer samples. Limited by the length of this paper, we choose to present the detailed results of the training and prediction set of narrow NN and LDA from case 1, and the results of the fine tree and bagged tree model from case 2 can be found in the supplementary file.

In general, it was concluded that concentrations of elements are important for good discrimination results. In multiple testing, the difference in classification model accuracy is very stable, with a standard deviation of less than 3%; hence, prediction accuracy was used as the comparison standard. The best prediction accuracy was provided by narrow and trilayer NN models for whole blood samples, and decision tree models for serum samples to differentiate between breast cancer and non-cancer. Therefore, in both cases, the blood samples could be clearly distinguished from the non-cancer by implementing the best prediction models and leading to an optimal ROC property. Other than sample preparation which typically took 5 to 7 minutes to prepare a sample, the total analyzing time was 2 seconds, including spectrum calculation and predictive classification results in each case. Our findings thus showed that LIBS is a potential diagnostic technique for the rapid diagnosis of breast cancer.

Indeed, our samples are not too much to summarize the regular pattern to find the statistical significance. *P* values are one of the most widely used concepts in statistical analysis to illustrate the statistical significance. For this purpose, *t*-test was used to determine the value of *p* as a statistical power to justify the number of samples used in this study. The obtained spectral data with feature line selection of five trace elements for both whole blood and serum samples showed significant variations because the p values in the statistical *t*-test were less than 0.05. Table. 5 shows the mean (M) and standard error (SE) values of training and prediction accuracy of all the classification model for different number of samples used for whole blood and serum samples. With two types of samples that are statistically different at *p* < 0.05 levels, the means in the same column are suitable values.

**Table 5.** Mean and standard error of training and prediction accuracy for both cases

Type of Sample	Sample distribution	Total No of samples (cancer + non-cancer)	M ± SE
**Case 1(Whole Blood)**	Training Set	17	0.765 ± 0.025
	Prediction Set	7	0.757 ± 0.018
**Case 2 (Serum)**	Training Set	25	0.745 ± 0.034
	Prediction Set	13	0.666 ± 0.021

The biggest challenge for LIBS in future studies is the homogeneity, heterogeneity and sensitivity of the sample surface as it will have an impact on the energy coupling and plasma excitation for biological samples. The characteristics of laser-matter interaction are influenced by the inhomogeneity of samples and the LIBS system fluctuation. By comparing the atomic emission lines obtained from the whole blood and serum, this work serves as an initial study that illustrates how the LIBS approach can be used to differentiate between different types of blood samples. To improve LIBS spectral signals and make them more applicable to a large number of human samples, further investigation into the blood is still required.

## 5. Conclusions

In this preliminary research, an efficient and fast method is proposed for early diagnoses of human breast cancer with a comparison of whole blood and serum samples using LIBS combined with machine learning models for the first time. 11 atomic emission lines of five elements Ca, N, C, Na and CN-band from breast cancer and non-cancer samples were used for the discrimination analysis. PCA results of LIBS spectral data were not differentiated clearly between cancer and non-cancer samples. Moreover, machine learning models including decision trees, discrimination analysis, logistic regression, naïve byes, support vector machine, k-nearest neighbor, ensemble, and neural networks classifiers were applied for classification between whole blood and serum samples of breast cancer and non-cancer. In the case of whole blood samples, narrow neural networks and trilayer neural networks both provided 91.7% highest prediction accuracy and in the case of serum samples, all the decision tree models provided 89.7% highest prediction accuracy. For case 1, Linear discrimination analysis and neural network models provided the best option for training and class prediction. Meanwhile, for case 2, decision trees and bagged trees gave the best option for training and class prediction. These outcomes demonstrated that the suggested technique can be a useful tool for quick initial screening of breast cancer. Furthermore, using whole blood as sample achieved strong emission lines of spectra, better discrimination results of PCA and maximum prediction accuracy of machine learning models as compared to using serum samples. With these merits, it is logical to argue that whole blood samples could be a good option for the rapid detection of breast cancer. This research on blood shows a high detection sensitivity emphasizing the clinical applications of this technique. We will carry out an additional study based on these results to support LIBS applications in the biomedical industry by identifying a large number of blood samples. Meanwhile, more research is needed to raise the standard of diagnosis of cancer by using LIBS.

## Data Availability

Data underlying the results presented in this paper are not publicly available at this time but may be obtained from the corresponding author upon reasonable request.

## Supplemental document

See Supplement 1 for supporting content.
