# Supplementary material for: Comparison of whole blood and serum samples of breast cancer based on laser-induced breakdown spectroscopy with machine learning
Source: Biomed Opt Express. 2023 May 3;14(6):2492–509. doi: 10.1364/BOE.489513 (PMC10278612; doi:10.1364/BOE.489513)
Supplement: Supplementary file 1 [file boe-14-6-2492-s001.pdf]

Supplemental Document

Biomedical Optics EXPRESS

Comparison of whole blood and serum samples of breast cancer based on laser-induced breakdown spectroscopy with machine learning: supplement

Bushra Sana Idrees,[1,2] **Geer Teng,**[1,3,9] **Ayesha Israr,**[4] **Huma Zaib,**[4] **Yasir Jamil,**[4] **Muhammad Bilal,**[5,6] **Sajid Bashir,**[7] **M. Nouman Khan,**[1,2] **and Qianqian Wang**[1,2,8,10]

[1] *School of Optics and Photonics, Beijing Institute of Technology, Beijing 100081, China*
[2] *Key Laboratory of Photonic Information Technology, Ministry of Industry and Information Technology, Beijing Institute of Technology, 100081 Beijing, China*
[3] *Department of Engineering Science, Institute of Biomedical Engineering, University of Oxford, Oxford, OX3 7LD, United Kingdom*
[4] *Laser Spectroscopy Lab, Department of Physics, University of Agriculture Faisalabad, 38090, Pakistan*
[5] *Institute of Engineering Thermophysics, Chinese Academy of Sciences, Beijing 100190, China*
[6] *University of Chinese Academy of Sciences, Beijing 100049, China*
[7] *Punjab Institute of Nuclear Medicine Hospital, Faisalabad 2019, Pakistan*
[8] *Yangtze Delta Region Academy of Beijing Institute of Technology, Jiaxing 314033, China*
[9] *geer.teng@eng.ox.ac.uk*
[10] *qqwang@bit.edu.cn*

This supplement published with Optica Publishing Group on 3 May 2023 by The Authors under the terms of the Creative Commons Attribution 4.0 License in the format provided by the authors and unedited. Further distribution of this work must maintain attribution to the author(s) and the published article's title, journal citation, and DOI.

Supplement DOI: https://doi.org/10.6084/m9.figshare.22649851

Parent Article DOI: https://doi.org/10.1364/BOE.489513

Comparison of whole blood and serum samples of Breast Cancer based on Laser-Induced Breakdown Spectroscopy with Machine Learning

In case 1, narrow NN and LDA showed the best prediction results. In narrow neural network, the training set accuracy was 92.2%, a false positive rate of 8.3% of cancer samples were missclassified and 5 spectra from first and 4 spectra from second showed variation from missclassified samples. However, a false negative rate of 7.3% of non-cancer samples were identified as cancer samples and 4 spectra from first and 3 spectra from second showed variation from missclassified samples. It resulted in a specificity of 92.7% and sensitivity of 91.7% respectively. All cancer and non-cancer samples were completely classified. Only two cancer was mistakenly identified, and two non-cancer was misclassified as cancer. Prediction accuracy results of Narrow NN can found in paper. Fig. S1 shows the confusion matrix of narrow NN produced from training model.

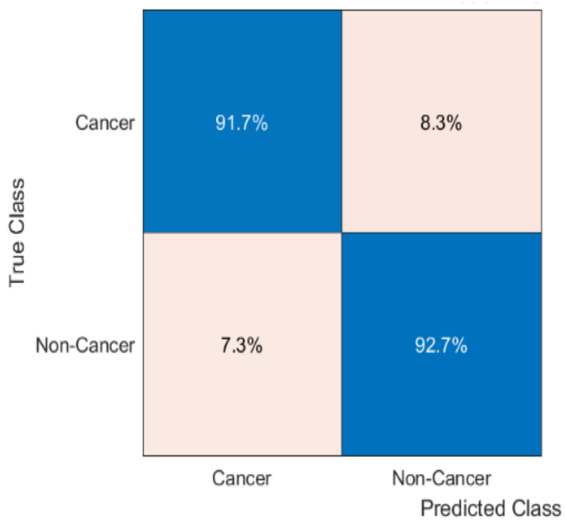

Fig. S1. Shows the confusion matrix of the narrow neural network produced from the training model for discriminating the whole blood samples of breast cancer versus non-cancer.

Similarly, LDA also showed the best prediction results. In LDA, the training set accuracy was 88.2% and a false positive rate of 10.2% of cancer samples were missclassified and 5 spectra from first, 4 spectra from second and 2 from third showed variation from missclassified samples. However, a false negative rate of 13.5% of non-cancer samples were identified as cancer samples and only 7 spectra showed variation from a single misclassified sample. It resulted in a specificity of 86.5% and sensitivity of 89.8% respectively. All cancer and non-cancer samples were completely classified. Only three cancer was mistakenly identified, and one non-cancer was misclassified as cancer. Prediction accuracy results of LDA was 90.5% and a false positive rate of 6.2% of cancer samples were missclassified and 5 spectra showed variation from a single missclassified sample. It resulted in a specificity of 86.1% and sensitivity of 83.8% respectively. Almost all cancer and non-cancer samples were completely classified. Fig. S2 shows the confusion matrix of LDA produced from (a) training model and (b) prediction model.

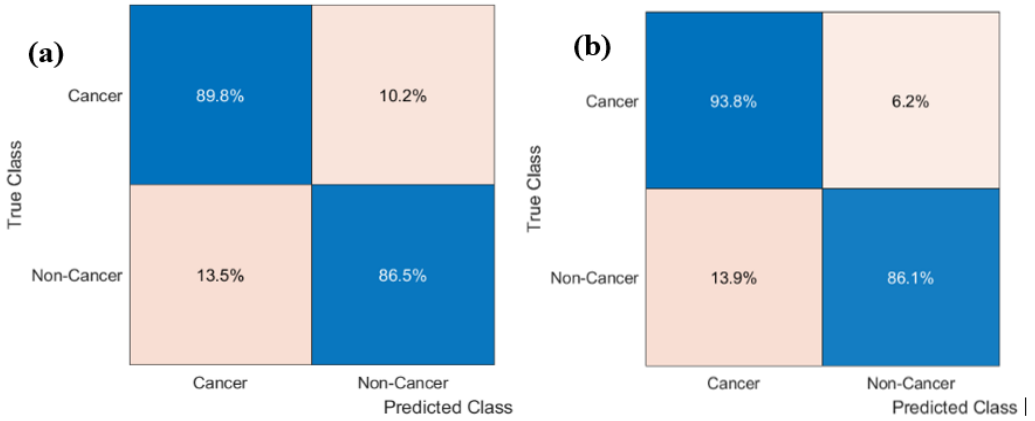

Fig. S2 shows the confusion matrix of LDA produced from (a) training model and (b) prediction model from case 1.

In case 2, Fine tree and bagged tree showed the best prediction results. In fine tree, the training set accuracy was 95.0%, and a false positive rate of 5.6% of cancer samples were missclassified and 6 spectra from first and 2 spectra from second showed variation from missclassified samples. However, a false negative rate of 4.5% of non-cancer samples were identified as cancer samples and only 7 spectra showed variation from a single misclassified sample. It resulted in a specificity of 95.5% and sensitivity of 94.4% respectively. All cancer and non-cancer samples were completely classified. Only two cancer was mistakenly identified, and one non-cancer was misclassified as cancer. Prediction accuracy detailed results of fine tree can be found in paper. Fig. S3 shows the confusion matrix of fine tree produced from training model.

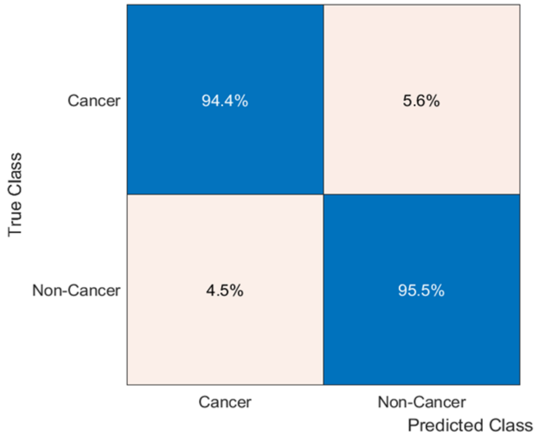

Fig. S3 shows the confusion matrix of fine tree produced from training model from case 2.

Bagged tree also showed the best training and prediction results. The training set accuracy was 96.0% and a false positive rate of 5.6% of cancer samples wa6 missclassified and 5 spectra from first and 2 spectra from second showed variation from missclassified samples. However, a false negative rate of 2.6% of non-cancer samples were identified as cancer samples and only 4 spectra showed variation from a single misclassified sample. It resulted in a specificity of 97.4% and sensitivity of 94.4% respectively. All cancer and non-cancer samples were completely classified. Only two cancer was mistakenly identified, and one non-cancer was misclassified as cancer. The prediction set accuracy was 84.6% and a false positive rate of 27.8% of cancer samples were missclassified and total 20 spectra showed variation from four different missclassified samples. However, a false negative rate of 4.8% of non-cancer samples were identified as cancer samples

and only 4 spectra showed variation from a single misclassified sample. It resulted in a specificity of 95.2% and sensitivity of 72.2% respectively. All cancer and non-cancer samples were completely classified. Only four cancer was mistakenly identified, and one non-cancer was misclassified as cancer. Prediction accuracy results of fine tree can be found in paper. Fig. S4 shows the confusion matrix of bagged tree produced from (a) training and (b) prediction model.

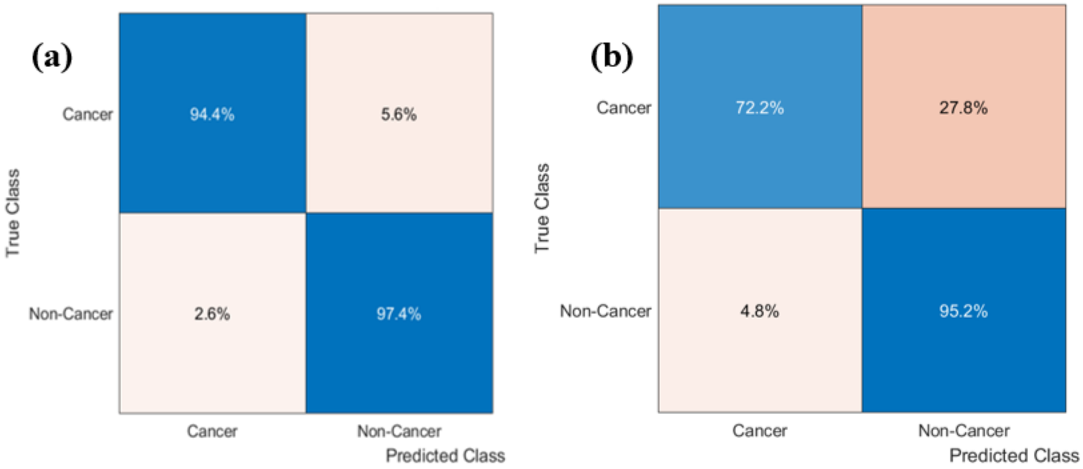

Fig. S4 shows the confusion matrix of bagged tree produced from (a) training model and (b) prediction model from case 2.
